# Dental Education

**Published:** 1890-09

**Authors:** J. H. Grant

**Affiliations:** Palestine, Texas.


ARTICLE II.

### DENTAL EDUCATION.

DELIVERED BEFORE THE TEXAS DENTAL ASSOCIATION 1890,
BY DR. J. H. GRANT, OF PALESTINE, TEXAS.

Education is one and the same thing, whether it pertains to a profession or trade. It is that acquired knowledge which brings man in contact with his fellow man in a high degree of culture, refinement and morals. The benefits of education are not confined alone to the person educated.

Education is the very root of heredity. The sharpened intelligence, strengthened faculties and improved morals which it gives are an estate that is transmitted by blood from parent to child in obodience to an immutable law of nature.

Its beneficial results are a property, which by the law of natural transmission, becomes a fixed estate in a man's posterity, and of which he cannot be deprived, either by legislative enactment, judicial fiction or any man's fraud or dishonesty.

The great scholar, Gibbon, has told us that every person has two educations, one which he receives from others and one more important which he gives himself.

And thus we see that our annual Dental meetings have a responsibility resting upon them to-day as a profession, to advance the younger men to that degree of scientific knowledge and practical skill, that will not only claim the attention of the medical profession at large, but claim the attention of the scholars of the world. We have, it is true, able writers, learned professors and skilled operators, with perhaps, a fair proportion of members of respectable talents and attainments, and yet we know and realize that there are many in our ranks who are deficient in the attainments and skill necessary to bring them up to that standard we should desire.

And if as a profession, we the members of this Association, strive to do what I conceive to be our duty and that, that the Chairman of your Executive committee has suggested, we will bring our meetings up to that degree of scientific proficiency that will cause our meetings to rank, as a post-graduate course, lasting for all time to come. It is in our annual meeetings that the devices for one member suggests ideas to another that stimulate him to further research and experiment. Our profession has made great advances in dental science and learning in the last thirty years and I will admit that the knowledge is not seen only in the principles and practice of the profession, but also in the character and standing of its members.

It has not been a great while since a dentist was thought to require no other talent and skill than that of a a country barber, but to day it stands risen to the dignity of a profession equal to that of any other, and which to be successfully followed demands long and careful study, thorough knowledge and practical skill, superior in some respects to any other profession.

It is true that quacks are common to all professions and vocations, but they are more degrading to dentistry because the public have no certain means of distinguishing between the qualified and those who are not, and I regret to say that it appears that the time has come when a man must be estimated by the printer's ink he displays in his daily papers or barn-door posters scattered on the streets, calling special attention to different grades of work, and many instances superior classes of work, and all work guaranteed. Must we always submit to such quackery and imposition? I hold that it is an imposition, because the credulous are imposed upon in many instances to their discomfort and sorrow. If I was the president of a dental college, I pledge you my word, I would require every graduate earning his diploma to obligate himself on receiving it not to pull his alma mater down, by resorting to such quackery and charlatanism. I dislike to be arrogant or exclusive; it is desirable and inevitable that there should be a wide difference in the standing of dentists in any community.

The great question of to-day is how are we to remedy this? It can only be done by compelling every one who comes to our state to present a certificate of identity with the profession including his diploma, or stand an examination before their board who should have the authority to cause the arrest of any man who should resort to such advertisements as are often seen on our streets and in our daily papers. I believe in legitimate advertising in keeping with the dignity of any profession, but this wild-cat style some have I do condemn, and I think the better element in a profession always will. In this connection I will also speak of the district boards. The law as it is, we all know to be defective, but lame as it is, it is some help to us. While it admits a great many that are not thoroughly qualified under the preceeding clause, it gives us as members of these boards, when a man comes to locate who has no diploma, the authority to submit a rigid examination, to ascertain his qualifications, and in order to maintain and encourage the dignity of our profession in Texas, especially. If you agree with me, in increasing our membership and elevating our meetings to that standard that they stand as a post-graduate course, will say, that these district boards should be capable of examining an applicant on anatomy, physiology, pathology and surgery, indicating an examination entitling those who can creditably sustain themselves there into professional rank.

Then if in the departments of materia medica chemistry, operative and mechanical dentistry, the examinations are equally rigid, we may have no fear that a man willing to stand such an examination and who does stand it should feel proud to enter our ranks. You may think that I require too much of a student before he be allowed to practice our specialty. Do you not agree with the profession at large in wanting to elevate the standard of dentistry? If you do, then do you not say that the student who would practice our specialty should have a respectable knowledge of the bones, their most important parts and articulations, the names and positions of the principle arteries, veins and nerves, the form and regulation of the head, chest and abdomen, and of the structure and properties of the principal tissues, of the functions of digestion, absorption, circulation, respiration, secretion, motion and sensation.

The surgical anatomy and physiology of the organs of mastication, deglutition, taste and articulation, or inflammation and its consequences, of the healing of wounds, of the method of arresting hemorrhage, of the union of fractures, of the administration of anesthetics and the treatment of threatened death from their employment; of the diseases and injuries of the mouth, jaws fauces, and adjacent parts.

If the standard of qualification in these branches alluded to and especially in the mechanics both of office and laboratory are correspondingly high, there is no reason why such candidate should not be entitled to practice our specialty, known as dental surgery, and the qualifying of of young men after this manner is a sure way of introducing material that will always take a pride in elevating our profession and attending our meetings.

If we are ambitious and desire to elevate the standard of dentistry and our meetings above that usually accorded them, we must confine ourselves to closer study of the oral lessons and thereby through special concentration acquire proficiency in the diagnosis and treatment of all abnormal conditions of the mouth.

We all know that specialism is the inevitable outcome of the extension of medical art and science, and it is also acknowledged that it is impossible for any one man to master medical and surgical practice in all of its details. The wonderful advances which have been made in oral and ophthalmic surgery by dentists and specialists in other branches of medicine and surgery have compelled both popular and professional recognition.

The variety and importance of dental lesions demand a special and thorough preparation, special opportunities and special practice, in order that the highest results in their treatment may be secured.

It should not be that a mere compliance with arbitrary rules and attendance at the lectures of a college, with only a superficial examination at the close of the session, are requisite to qualify a condidate to receive his diploma, or practice upon his own responsibility.

No one should be allowed to receive a degree upon superficial knowledge. The applicant must earn his degree, and his title to it should be tested by a full and exhaustive examination in all the branches of knowledge with which a dentist should be familiar, for the successful practice of his profession and for the maintainance of an honorable position as a professional upright man.

I hold, however, that any one, who by his industry and perseverance has qualified himself to pass a rigid and satisfactory examination without the aid of colleges, is entitled to take rank in any profession equal to those who more favored by circumstances have been able to avail themselves of the important and to many, the necessary aid, which the colleges afford.

You may take our colleges of to-day or the office of a private preceptor if you please, and have either with a proper organization.

A course of study which carries the student progressively and systematically from one subject to another in a just and natural order and which is followed by minute examinations, that enforces a mental discipline, and which is of incalculable benefit to the student, and one that insures a thorough training in certain branches of dental instruction which could not be acquired under any other system.

This training is of special importance in the early years of a student's life and paves the way for the work he has before him in after practice. And the time is not far distant, when every dentist will have to acquire that knowledge of these studies already mentioned before he be allowed to practice, in order that he may be able to impart such knowledge to his patients and those he is brought in daily contact with.

There is a great demand to-day upon the profession for a higher order of acquaintance with different pathological conditions, and particularly in correct diagnosis, hence we—and by we (I mean colleges, private preceptors and dental meetings) must bring educated men into the profession both in literature and science, with minds that can make the fingers work out the thoughts of the brain, not merely a scientific knowledge or dexterous workmanship, however important both may be, but educated labor is the demand, and is this met?

I regret to say it is not. Our dental journals annually state that the yearly additions to the profession does not fall short of six hundred persons. Of this number only about one-third are graduates of respectability.

Now, then, I would ask how can this educated labor be attained? If not in dental colleges, can it be acquired from a private preceptor?

It cannot in the full sense of the word, for the reason that the private preceptor cannot make each department of dental science a particular and constant study. If delicate manipulation is desired, perfection in mechanical work must necessarily receive a limited attention.

And another obstacle is, that he has not the conveniences for illustrating and demonstrating that important knowledge in anatomy chemistry, physiology and pathology, an acquaintance with which is as essential to a dental practitioner for the basis of a sound practice as it is for him to discriminate between a cut artery and a cut vein, when called to arrest a hemorrhage.

In conclusion I cannot refrain from speaking of one other evil that is unfortunate in our dental colleges as well as in the medical colleges, which is to-day lowering the status of all professions, and that is, the cheap grade of instruction imparted at our dental colleges, by a spirit of rivalry, the mania that has sprung up in our profession of starting new dental colleges can only be attended with injurious consequences. This, if continued, will tend to degrade the standing of granting a diploma, the bad effects of some we have already seen, by the exposure made by these short-lived institutions in the way of bestowing honors.

The profession does not need more colleges, it needs less in number and a better quality. The faculties of these could then be composed of the best talent and pecuniarily sustained. Able instructors could then devote their whole time and attention to the duties devolving upon them, and the results would show themselves; results that as dentists proud of our profession we should uphold and encourage and insist that every young man receiving a beneficiary certificate from this Association, not only attend the first course, but obligate himself to obtain his degree.

If this is done we can in a measure, do away with a superficial knowledge only acquired, and demand a thorough course both in theory and practice, that will compel recognition to the advancement already made and that has characterized our profession during the last half century. —*Texas Dental Journal.*

